# Highly-selective µ-opioid receptor antagonism does not block L-DOPA-induced dyskinesia in a rodent model

**DOI:** 10.1186/s13104-020-04994-7

**Published:** 2020-03-12

**Authors:** Mitchell J. Bartlett, Lisa Y. So, Lajos Szabò, David P. Skinner, Kate L. Parent, Michael L. Heien, Todd W. Vanderah, Robin Polt, Scott J. Sherman, Torsten Falk

**Affiliations:** 1 Department of Neurology, The University of Arizona, Tucson, AZ 85724 USA; 2 Department of Pharmacology, The University of Arizona, Tucson, AZ 85724 USA; 3 Graduate Interdisciplinary Program in Neuroscience, The University of Arizona, Tucson, AZ 85724 USA; 4 Department of Chemistry & Biochemistry, The University of Arizona, Tucson, AZ 85721 USA

**Keywords:** Mu-opioid receptors, Delta-opioid receptors, Basal ganglia, Parkinson’s disease, Levodopa

## Abstract

**Objectives:**

Dopamine-replacement utilizing L-DOPA is still the mainstay treatment for Parkinson’s disease (PD), but often leads to development of L-DOPA-induced dyskinesia (LID), which can be as debilitating as the motor deficits. There is currently no satisfactory pharmacological adjunct therapy. The endogenous opioid peptides enkephalin and dynorphin are important co-transmitters in the direct and indirect striatofugal pathways and have been implicated in genesis and expression of LID. Opioid receptor antagonists and agonists with different selectivity profiles have been investigated for anti-dyskinetic potential in preclinical models. In this study we investigated effects of the highly-selective μ-opioid receptor antagonist CTAP (> 1200-fold selectivity for μ- over δ-opioid receptors) and a novel glycopeptide congener (gCTAP5) that was glycosylated to increase stability, in the standard rat LID model.

**Results:**

Intraperitoneal administration (*i.p.*) of either 0.5 mg/kg or 1 mg/kg CTAP and gCTAP5 completely blocked morphine’s antinociceptive effect (10 mg/kg; *i.p.*) in the warm water tail-flick test, showing in vivo activity in rats after systemic injection. Neither treatment with CTAP (10 mg/kg; *i.p.*), nor gCTAP5 (5 mg/kg; *i.p.*) had any effect on L-DOPA-induced limb, axial, orolingual, or locomotor abnormal involuntary movements. The data indicate that highly-selective μ-opioid receptor antagonism alone might not be sufficient to be anti-dyskinetic.

## Introduction

The mainstay of treatment for Parkinson’s disease (PD) consists of dopamine replacement therapy with levodopa (L-DOPA; l-3,4-dihydroxyphenylalanine), which was introduced in the 1960′s, remains the most efficacious symptomatic therapy and is considered the gold standard. Although tolerated well in the short term, the chronic use of levodopa, in combination with progression of the disease, results in the development of involuntary movements termed L-DOPA-induced dyskinesia (LID), a common and disabling side effect [1, 2]. Individuals with LID exhibit abnormal levels of many neurotransmitters in addition to dopamine. For example, endogenous opioids are dysregulated in dyskinetic patients, and the striatum is rich in receptors for µ- and δ-opioid receptors. Striatal neurons utilize the opioid peptides dynorphin and met-enkephalin as co-transmitters, and levels of these peptides are altered significantly in PD. Long-term L-DOPA therapy leading to the development of LID elevates levels of opioid peptides and mRNA encoding their precursors in animal models of PD. In postmortem studies, individuals with PD who expressed motor fluctuations due to long-term L-DOPA use express increased striatal preproenkephalin A and preproenkephalin B levels [3, 4]. There is disparity of data from both preclinical and clinical studies that has led to conflicting concepts that opioids represent either a cause of LID or a compensatory mechanism, and both opioid receptor antagonism and agonism, possibly specific to the μ-opioid receptors, are actively being studied for their anti-dyskinetic properties [5–7].

In order to investigate the anti-dyskinetic potential of specifically only blocking μ-opioid receptors we conducted experiments in the standard rodent model of established LID, 6-hydroxydopamine (6-OHDA)-lesioned hemi-parkinsonian rats primed with L-DOPA, using the highly-selective μ-opioid receptor antagonists CTAP (IC50 = 3.5 nM and > 1200-fold selective for μ- over δ-opioid receptors) [8] and a congener gCTAP5, that had been glycosylated to increase stability [9].

## Main text

### Methods

#### Material

CTAP glycopeptides were prepared using published methods [10]. **CTAP**: *d*Phe-Cys-Tyr-*d*Trp-Arg-Thr-Pen-Thr (parent peptide); glycosylated congener **gCTAP5**: *d*Phe-Cys-Tyr-*d*Trp-Arg-Thr-Pen-Thr-Gly–Gly-Ser-β Glucopyranoside.

#### Peptide assembly

The peptides were assembled on Rink resin using Fmoc (fluorenylmethoxycarbonyl protecting group) methodology. Couplings were accomplished with 1-hydroxy-benzotriazole, *N*,*N′*-diisopropyl-carbodiimide and the desired amino acid. Coupling times ranged from 40 min up to 4 h for the more sterically encumbered sequences. For the serine glucoside, 1.3 equiv of the amino acid, and the couplings were monitored with the Kaiser ninhydrin test. For all other amino acids, 3.0 equiv were used. For tyrosine and cysteine, penicillamine O-tert-butyl and S-trityl protecting groups were employed. Then, the N-terminal FMOC-groups were removed, and the acetate groups of the glycoside were removed with hydrazine hydrate in MeOH while on the solid support. After ester cleavage, the resin was washed several times to remove excess hydrazine and vacuum-dried to obtain mass determination. The dried peptide resins were cleaved with a cocktail mixture (9.0 mL trifluoroacetic acid (TFA), 1.0 mL CH_2_Cl_2_, 0.25 mL Et_3_SiH, 0.25 mL H_2_O, and 0.05 mL anisole per 1.0 g peptide resin) for 2 h at RT. After cleavage was complete, the solutions were filtered to remove the cleaved resin, and the resulting solution was concentrated to an oil *in vacuo*. After concentration, cold Et_2_O was poured over the peptide solutions for precipitation. The crude peptides were centrifuged, dried, and purified via preparative reverse-phase high performance liquid chromatography (HPLC) with a linear gradient of 5–80% CH_3_CN:0.1% aqueous TFA to yield the pure reduced compounds. These samples were cyclized with a solution of 3.0 equiv of K_3_Fe(CN)_6_ at pH 8.5 for roughly 16 h using a high-dilution, reverse-addition protocol. Then, these solutions were acidified to pH 4.0, anion-exchanged with Amberlite IRL-80 exchange resin, filtered, and lyophilized. The crude cyclic material was repurified with a preparative as before. **CTAP**: 63 mg (yield 8.7%), purity > 99% at 280 nm, retention time (Rt): 10.55 min (gradient 5–80%/15 min). Calc. C_51_H_69_N_13_O_11_S_2_ Mw: 1103.5, Found electrospray ionization (ESI) [M + H]^+^ 1104.6. **gCTAP5**: 47 mg (yield 6.4%), purity > 99% at 280 nm, Rt: 10.07-min (gradient 5–80%/15 min). Calc. C_64_H_90_N_16_O_20_S_2_ Mw:1466.6, Found ESI [M + H]^+^ 1467.3.

#### Animals for LID model and Tail flick experiments

Male Sprague–Dawley rats (*n *= 8 for LID study: 250 g and *n *= 8 for Tail Flick: 200 g; Harlan, Indianapolis, IN) were used and housed in a temperature and humidity-controlled room with 12-h light/dark cycles with food and water available ad libitum. All animals were treated as approved by the Institutional Animal Care and Use Committee at the University of Arizona and in accordance with the NIH Guidelines for the Care and Use of Laboratory Animals. Number of animals used and their suffering were minimized.

#### Tail-flick paradigm

The animals (*n *= 8) were gently restrained and nociception was administered by dipping the distal third of the tail in a 52 °C water bath. Latency to tail-flick was recorded as the time required for the tail to withdraw from the bath, with a cutoff of 10-seconds to prevent tissue damage. Prior to administering compounds, three measurements of tail-flick latency were recorded with 2-min intervals between tests to establish control latency. The antinociceptive effect of intraperitoneal (*i.p.*) morphine (10 mg/kg) was determined for each animal every 15 min with measurements of tail-flick latency between 15 and 90 min post-injection. The individual abilities of CTAP and gCTAP5 (0.1, 0.5 and 1 mg/kg, *i.p.*) to antagonize the antinociceptive effect of morphine were then tested at 48 h intervals. The μ-opioid receptor antagonists were administered 10-min after morphine in order to coincide with its peak effect. Latency time and maximum possible effect (%MPE) were calculated at 45 min post μ-opioid receptor antagonists injection.  %MPE = (post injection latency-baseline latency)/[cutoff (10 s)-baseline latency] × 100.

#### The unilateral 6-hydroxydopamine (6-OHDA)-lesion rat PD model

Injection of 20 μg 6-OHDA (5.0 μg/μl in 0.9% sterilized saline with 0.02% ascorbic acid; Sigma, St Louis, MO) in 2 locations in the medial forebrain bundle (MFB), as published [11, 12]. The rate of the injection was 0.5 μl per min using a Stoelting microinjector (Stoelting Co., Wood Dale, IL). Rats were pretreated 30 min prior with 12.5 mg/kg desipramine hydrochloride (Sigma, St Louis, MO) given *i.p.* to prevent damage to noradrenergic neurons.

#### Induction of LID in unilateral lesioned rats

Two weeks after surgery the unilateral 6-OHDA-lesioned rats were injected with D-amphetamine (5.0 mg/kg, *i.p.* injection; Sigma) to induce asymmetrical dopamine release. The number of ipsiversive rotations during 1-minute intervals, every 5 min were counted for a total of 60 min after the injection. 2) Rats with ≥ 4 rotations/minute were selected and were daily treated with 7 mg/kg L-DOPA (with 15 mg/kg benserazide, both *i.p.*; Sigma) for 3 weeks to establish LID, and the 6 rats that developed stable LID were included in the LID study.

#### Behavioral analysis in the LID rat model

L-DOPA-induced abnormal involuntary movements (AIMs) were scored by an experimentally blinded investigator according to an established protocol [11, 12], with a ‘within subjects cross over design’ to have a vehicle control for every drug tested. For each drug animals were randomized to either receive vehicle or drug on one testing day, and switched 3–4 days later, so that each drug has a separate vehicle control. For quantification of the severity of the AIMs, rats were observed individually in their standard cages every 20th min at 20–80 min after an injection of L-DOPA, and were classified as described [11, 12]. The sum of limb, axial, and orolingual (LAO) AIMs and the sum of locomotor AIMs scores per testing session were used for statistical analyses.

#### Euthanasia and brain tissue harvest

Rats were sacrificed after the last dose of drug with carbon dioxide. For a quantitative measure of the extent of the PD-lesion for *n *= 6 animals the whole brains were extracted, striatal tissue was prepped and frozen at − 80 °C. To validate the lesions post hoc quantitative dopamine (DA) measurements in striatal tissue was then conducted with HPLC-EC, as published [11, 12].

#### Data analysis

Statistical analysis was performed using GraphPad Prism 8.1 software (GraphPad Software, Inc., La Jolla, CA). Repeated measures one-way ANOVA with Tukey post hoc tests was used for the tail-flick time point raw MPE data. For the DA analysis a two-tailed *t* test of the raw data was conducted. Non-parametric Kruskal–Wallis test with Dunn’s multiple comparisons post hoc tests was used to compare the effect of treatment on LAO- and locomotor AIMs. The null hypothesis was rejected when *p* < 0.05.

### Results

**Fig. 1:**
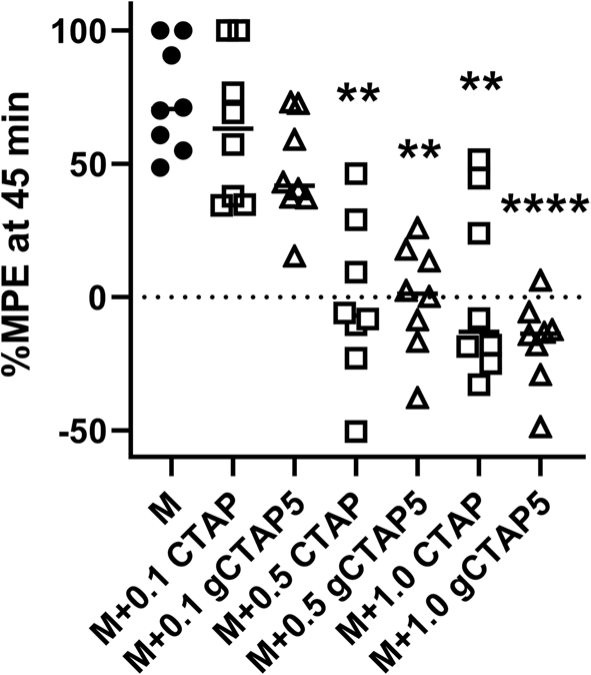
CTAP and gCTAP5 block morphine effects in a rat model of nociception. At the doses of 0.5 mg/kg and 1.0 mg/kg both gCTAP5 (open triangles) and CTAP (open squares) completely blocked the antinociceptive effect of morphine (10 mg/kg; black circles) effect at the maximum at 45 min. For gCTAP5 even at 0.1 mg/kg a trend of a reduction was evident that did not reach significance. The mean  %MPE ± SEM is plotted (*n *= 8; repeated measures ANOVA with Tukey post hoc tests; **p < 0.01, ****p < 0.0001)

CTAP had been shown in prior work to be a blood–brain barrier penetrant drug [8], and used in many rodent studies. Tail-flick testing verified that not only CTAP but also gCTAP5 crossed the blood–brain barrier sufficiently to exhibit a strong reduction of morphine’s antinociceptive activity, as tested with the warm water tail-flick paradigm (Fig. 1). This block of morphine’s maximal effect at 45 min was seen at doses as small as 0.5 mg/kg, *i.p.* for both CTAP and gCTAP5 (F_3.122, 21.85_ = 17.24; *p *< 0.0001).

**Fig. 2:**
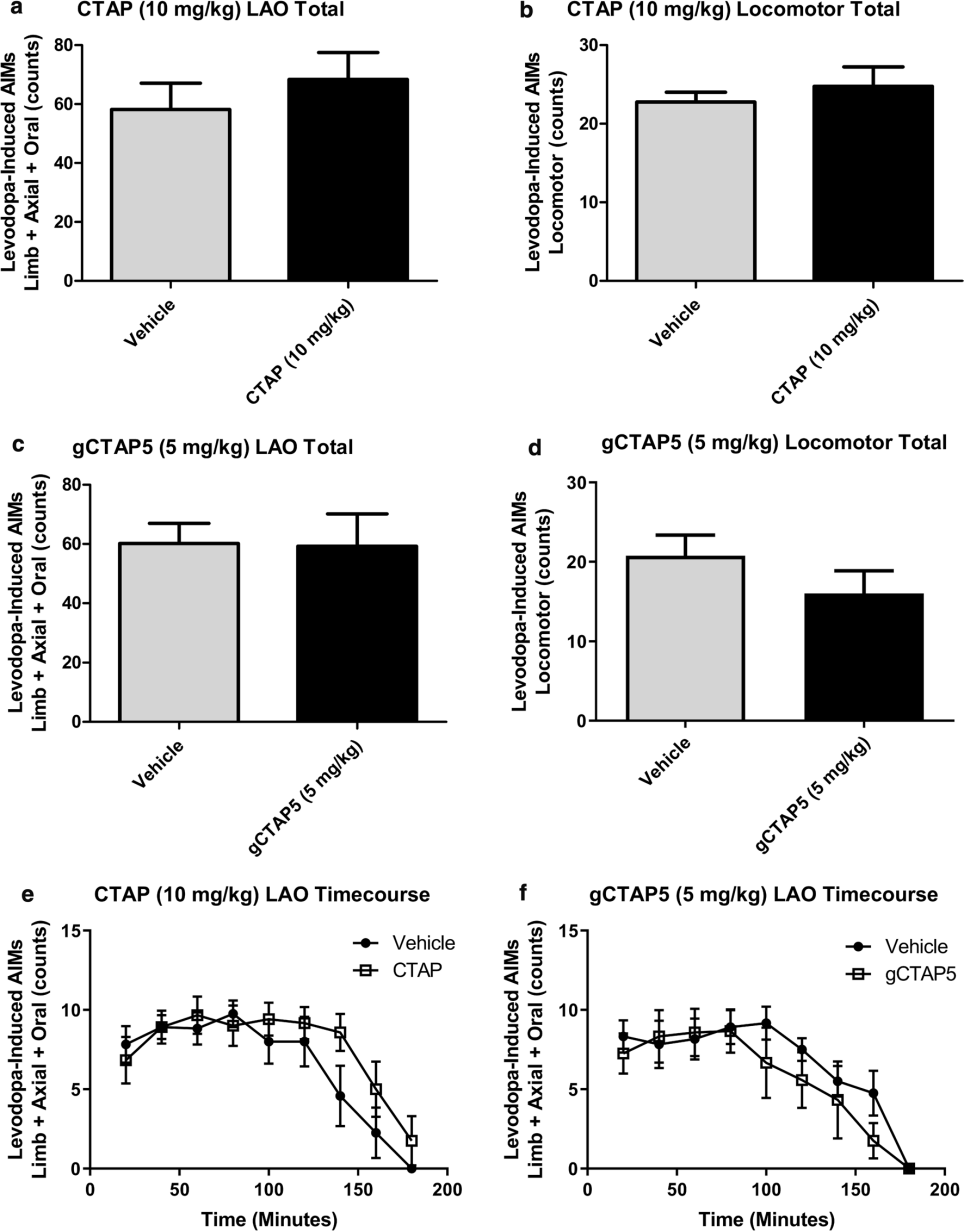
Highly-selective μ-opioid receptor antagonism had no effect on L-DOPA-induced AIMs. CTAP (10 mg/kg, *i.p.*) had no effect on either LAO or locomotor AIMs. **a** The mean total LAO AIMs scores over 180 min were plotted and there was no difference between vehicle (gray bar) and CTAP (black bar) condition (mean AIMs count ± SEM; *n *= 6; paired two-tailed *t*-test). **b** The mean total locomotor AIMs scores over 180 min were plotted and there was no difference between vehicle (gray bar) and CTAP (black bar) condition. Similarly, gCTAP5 (5 mg/kg, *i.p.*) had no effect on either LAO or locomotor AIMs. **c** The mean total LAO AIMs scores over 180 min were plotted and there was no difference between vehicle (gray bar) and gCTAP5 (black bar) condition. **d** The mean total locomotor AIMs scores over 180 min were plotted and there was no difference between vehicle (gray bar) and gCTAP5 (black bar) condition. In all graphs the mean AIMs counts ± SEM were plotted; *n *= 6; paired two-tailed *t*-test. **e** Time course of the LAO-AIMs (mean ± SEM) data after CTAP administration presented in (**a)**. **f** Time course of the LAO-AIMs (mean ± SEM) data after gCTAP5 administration presented in (**c)**

As has been shown in prior work [11, 12] the MFB 6-OHDA-lesion protocol in our hands leads to > 95% depletion of striatal DA content on the lesioned side. We confirmed the lesion post hoc by measuring striatal DA content, and did see a significant reduction *(n *= 6; two-tailed *t*-test on raw data before normalization, *p* < 0.0005) in the lesioned hemisphere by > 95%. In line with this, amphetamine-induced ipsiversive rotations for PD animals (*n *= 6) included in this study was 5.1 ± 1.3 (mean ± SEM). These animals exhibited severe and stable LID after the 3-week priming with daily L-DOPA treatment. The stability was confirmed with testing the severity of the AIMs three times 3–4 days apart before testing of compounds ensued. The severity of LID is evidenced by mean LAO-AIMs scores of 60, and mean locomotor AIMs scores of 20. When testing for anti-dyskinetic activity in the LID rats, neither CTAP nor gCTAP5 did reduce either LAO-AIMs (Fig. 2a and c) or locomotor AIMs (Fig. 2b and d), not at 5 mg/kg gCTAP, or at the high dose of 10 mg/kg CTAP. The time course data for the LAO-AIMs for CTAP vs. vehicle and gCTAP5 vs. vehicle are presented in Fig. 2 e and f, respectively.

### Discussion

The data presented indicate that highly-specific μ-opioid receptor antagonism does not reduce LID in the standard rodent model. This was a surprising finding given prior published findings showing anti-dyskinetic activity of drugs with μ-opioid antagonist activity [7]. There could be ‘hidden’ beneficial δ-opioid receptor properties in the μ-opioid receptor antagonist ADL5510 that had not been examined as extensively as the much used highly-selective μ-antagonist CTAP. Specifically, it is important that ADL5510 is only 15-fold selective for μ- vs. δ-opioid receptors, while CTAP is > 1200 fold selective for μ- over δ-opioid receptors. In that respect, it is of interest that ADL5510 had a U-shaped dose–response curve in the non-human primate experiments, indicative of more than one property being involved in its therapeutic activity [7]. Further, a recent study did show that the novel molecule DPI-289, a mixed δ-opioid agonist/μ-opioid receptor antagonist, was anti-dyskinetic in both rodent and non-human primate LID models [13]. Similarly, the mixed κ-opioid receptor agonist/μ-opioid receptor antagonist nalbuphine did also reduce LID in dyskinetic non-human LID primates [14]. And the combination of the mixed μ/δ-opioid receptor agonist MMP-2200, with the *N*-Methyl-d-aspartate receptor (NMDAR) antagonist MK-801 was also shown to be anti-dyskinetic [12]. This points to a possible need of a contribution of μ- and δ-or κ-opioid receptor activity, or a combination of drugs to target opioid and NMDA receptors to achieve anti-dyskinetic action.

In Conclusion, combined with the presented data, those findings discussed above indicate that a combination of more than one opioid receptor activity might be needed for full anti-dyskinetic activity of therapeutic drug candidates targeting the opioid system to treat LID, and that highly-selective μ-opioid receptor antagonism alone is not sufficient.

## Limitations

Species differences could also account for the discrepancy to the published anti-dyskinetic effects of the modestly-selective μ-opioid receptor antagonist ADL5510 in a non-human primate model of LID [7]. A full dose–response was not conducted in the LID studies, therefore anti-dyskinetic effects of a lower dose cannot be ruled out, yet are not likely given that U-shaped response curves point to a second mode of action that is unlikely in the case of a compound as highly-selective as CTAP (> 1200-fold μ- vs. δ-opioid receptors) [8].

## Data Availability

The datasets used and/or analyzed during the current study are available from the corresponding author on reasonable request.

## Abbreviations

ADL5510: Modestly-selective µ-opioid receptor antagonist
AIMs: Abnormal involuntary movements
ANOVA: Analysis of variance
CTAP: Highly-selective µ-opioid receptor antagonist
DA: Dopamine
DPI-289: Mixed δ-opioid agonist/µ-opioid receptor antagonist
ESI: Electrospray ionization
Fmoc: Fluorenylmethoxycarbonyl protecting group
gCTAP5: Glycosylated CTAP
HPLC: High performance liquid chromatography
HPLC-EC: HPLC with electric coupling
IC50: Half maximal inhibitory concentration
*i.p*: Intraperitoneal administration
LAO: Limb, axial and orolingual
L-DOPA: l-3,4-Dihydroxyphenylalanine
LID: L-DOPA-induced dyskinesia
MFB: Medial forebrain bundle
MPE: Maximum possible effect
NMDAR: *N*-Methyl-d-aspartate receptor
PD: Parkinson’s disease
Rt: Retention time
SEM: Standard error of the mean
6-OHDA: 6-Hydroxydopamine
TFA: Trifluoroacetic acid

## References

[1] Olanow CW, Stern MB, Sethi K (2009). The scientific and clinical basis for the treatment of Parkinson disease. Neurology.

[2] Huot P, Johnston TH, Koprich JB, Fox SH, Brotchie JM (2013). The pharmacology of L-DOPA-induced dyskinesia in Parkinson’s disease. Pharmacol Rev.

[3] Gertler TS, Chan CS, Surmeier DJ (2008). Dichotomous anatomical properties of adult striatal medium spiny neurons. J Neurosci.

[4] Seizinger BR, Grimm C, Höllt V, Herz A (1984). Evidence for a selective processing of proenkephalin B into different opioid peptide forms in particular regions of rat brain and pituitary. J Neurochem.

[5] Breslin MB, Lindberg I, Benjannet S, Mathis JP, Lazure C, Seidah NG (1993). Differential processing of proenkephalin by prohormone convertases 1(3) and 2 and furin. J Biol Chem.

[6] Atwood BK, Kupferschmidt DA, Lovinger DM (2014). Opioids induce dissociable forms of long-term depression of excitatory inputs to the dorsal striatum. Nat Neurosci.

[7] Koprich JB, Fox SH, Johnston TH, Goodman A, Bourdonnec B, Dolle RE, DeHaven RN, DeHaven-Hudkins DL, Little PJ, Brotchie JM (2011). The selective mu-opioid receptor antagonist ADL5510 reduces levodopa-induced dyskinesia without affecting antiparkinsonian action in MPTP-lesioned macaque model of Parkinson’s disease. Mov Disord.

[8] Abbruscato TJ, Thomas S, Hruby VJ, Davis TP (1997). Blood-brain barrier permeability and bioavailability of a highly potent and mu-selective opioid receptor antagonist, CTAP: comparison with morphine. J Pharmacol Exp Ther.

[9] Jones EM, Polt R (2015). CNS active O-linked glycopeptides. Front Chem.

[10] Mitchell SA, Pratt MR, Hruby VJ, Polt R (2001). Solid-phase synthesis of O-linked glycopeptide analogues of encephalin. J Org Chem.

[11] Bartlett MJ, Joseph RM, LePoidevin LM, Parent KL, Laude ND, Lazarus LB, Heien ML, Estevez M, Sherman SJ, Falk T (2016). Long-term effect of sub-anesthetic ketamine in reducing L-DOPA-induced dyskinesias in a preclinical model. Neurosci Lett.

[12] Flores AJ, Bartlett MJ, Root BK, Parent KL, Heien ML, Porreca F, Polt R, Sherman SJ, Falk T (2018). The combination of the opioid glycopeptide MMP-2200 and a NMDA receptor antagonist reduced l-DOPA-induced dyskinesia and MMP-2200 by itself reduced dopamine receptor 2-like agonist-induced dyskinesia. Neuropharmacology.

[13] Johnston TH, Versi E, Howson PA, Ravenscroft P, Fox SH, Hill MP, Reidenberg BE, Corey R, Brotchie JM (2018). DPI-289, a novel mixed delta opioid agonist/mu opioid antagonist (DAMA), has L-DOPA-sparing potential in Parkinson’s disease. Neuropharmacology.

[14] Potts LF, Park ES, Woo JM, Dyavar Shetty BL, Singh A, Braithwaite SP, Voronkov M, Papa SM, Mouradian MM (2015). Dual κ-agonist/μ-antagonist opioid receptor modulation reduces levodopa-induced dyskinesia and corrects dysregulated striatal changes in the nonhuman primate model of Parkinson’s disease. Neurology.

